# Study of Fatigue Crack Initiation and the Propagation Mechanism Induced by Pores in a Powder Metallurgy Nickel-Based FGH96 Superalloy

**DOI:** 10.3390/ma17061356

**Published:** 2024-03-15

**Authors:** Shuang Yi, Shichao Zhang, Denghui Wang, Jianxing Mao, Zheng Zhang, Dianyin Hu

**Affiliations:** 1 School of Materials Science and Engineering, Beihang University, Beijing 100191, China; yishuang@buaa.edu.cn (S.Y.); zhangshichaomail@163.com (S.Z.); 18503437855@163.com (D.W.); 2 Research Institute of Aero-Engine, Beihang University, Beijing 100191, China; maojx@buaa.edu.cn (J.M.); hdy@buaa.edu.cn (D.H.); 3 Beijing Key Laboratory of Aero-Engine Structure and Strength, Beijing 100191, China; 4 United Research Center of Mid-Small Aero-Engine, Beijing 100191, China

**Keywords:** FGH96 superalloy, thermally induced pores, fatigue crack initiation and propagation, crack deflection

## Abstract

Thermally induced pores (TIPs) are generally the source of fatigue crack initiation in the powder metallurgy (PM) Ni-based FGH96 superalloy. The effect of TIPs on fatigue crack initiation on the surface of the FGH96 superalloy was detected using scanning electron microscopy (SEM). The cause of fatigue crack deflection was studied using electron backscatter diffraction (EBSD) analysis. The results indicated that there are two states of TIPs including isolated TIPs and clustered TIPs located at the grain boundary. The investigation of crack initiation and propagation around TIPs was conducted in detail through the comprehensive integration of experimental findings and computational results. For cracks initiated by isolated TIPs, the maximum equivalent size and the ratio of the vertical–parallel axis to the loading direction of the TIPs reveal a linear relationship, and both of them determine crack initiation. Regarding clustered TIPs, the constituent pores of the clustered TIPs will compete to initiate cracks based on the experimental results, and the largest pore will be more likely to initiate cracking. Moreover, the results showed that fatigue crack propagation can be hindered by hard-orientation grains and twins with a low Schmid factor (SF). Large-angle crack deflection due to twins with a low SF can significantly increase crack length and resistance to crack propagation.

## 1. Introduction

Ni-based superalloys derived by powder metallurgy (PM) methods are widely applied in the manufacture of aero-engine turbine discs as a favored structural material in the aerospace industry [1,2]. The superior creep, fatigue, and oxidation resistance performance of PM Ni-based superalloys at elevated temperatures can mainly be attributed to their firm structure [3,4,5]. Compared with conventional cast and wrought Ni-based superalloys, PM Ni-based superalloys can efficiently exterminate solidification segregation and promote alloying levels to fulfill the demand for better overall mechanical performance [6,7,8,9].

The three most common types of defects in PM superalloys are non-metallic inclusions (NMIs), prior particle boundaries (PPBs), and thermally induced pores (TIPs) [10,11]. It has also been shown that there are competing fatigue failure behaviors among defects [12,13,14]. The existence of internal defects such as micropores as well as twins/dislocations is a very usual problem for PM materials, in particular, for Ni-, Ti-, Al-, and Fe-containing alloys. The formation of TIPs can primarily be attributed to entrapped insoluble gas, insoluble gases, and leakage in the container [15,16,17]. As a result, the continuity of the matrix will be destroyed by TIPs.

The significant deleterious influences of TIPs on the mechanical performance of powder superalloys have been thoroughly examined. It has been observed that impact energy, tensile ductility, and creep-fatigue life are significantly reduced as a result of increased porosity in PM Ni-based superalloys [18,19,20,21]. TIPs may cause crack initiation and induce fatigue cracking failure during service [22,23,24]. Teschke et al. [25] noted that fatigue strength can be significantly affected by the size of the pores, and porosity is the crucial impact leading to failure. Kumar et al. [26] revealed the relationship between strength properties and the size of extreme-sized pores. Consequently, pores could greatly worsen the mechanical performance of powder metallurgy components, particularly their fatigue properties. Recently, more researchers have concentrated on the failure mechanisms of pores on PM superalloys. Le et al. [27] found that fatigue behavior depends on the spatial position of the defect to some extent. Meng et al. [28] investigated fatigue crack initiation and propagation behavior. The results indicated that the remaining pores at weakly bonded interparticle boundaries could extend under cyclic loading as well as trigger secondary cracks, decreasing the fatigue crack propagation rate.

Although these extensive above-mentioned studies deal with the influence of pores on mechanical performance in PM superalloys, the pore type is not regarded in any way. It is essential to illustrate the mechanisms of different types of pores and their effects on fatigue performance. Thus, detailed work and systematic analysis are essential for unravelling the role of different types of pores in crack initiation.

To fill this gap, this work attempts to illuminate the influence of different TIP types on fatigue crack initiation. Accordingly, the microstructure variations across different TIP types in the powdered FGH96 superalloy are characterized in this paper. Pore-induced crack initiation behaviors were studied and compared through fatigue tests to identify the basic initiation mechanisms underlying fatigue crack initiation, depending on the TIP types. Moreover, a corresponding electron backscatter diffraction (EBSD) analysis was conducted to investigate the crack deflection phenomenon, which originated from pores in the fatigue test.

## 2. Materials and Methods

### 2.1. Materials

A conventional second-generation powdered FGH96 superalloy used for engine turbine disks was investigated in this study. The turbine disk utilized in this study had not undergone operation. Consequently, the accumulated damage from the period of operation was not considered. Hot isostatic pressing was carried out to fabricate a turbine disk at 1120 °C/120 MPa/4 h. Then, standard heat treatment of the experimental material was implemented, including alloy solution treatment at 1150 °C for 2 h followed by an aging treatment at 760 °C for 10 h. The chemical composition and mechanical properties of the FGH96 superalloy are presented in Table 1 and Table 2, respectively. X-ray photoelectron spectroscopy (XPS) was used to investigate the chemical composition of the materials outlined in Table 1. The mechanical properties presented in Table 2 were determined using a tensile test according to GB/T 228.1-2010 [29].

### 2.2. Experimental Procedure

**Figure 1:**
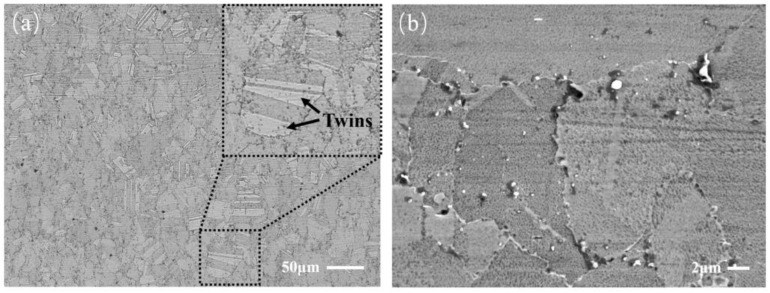
Microstructural observations of the FGH96 superalloy: (**a**) optical micrograph; (**b**) SEM image.

Microstructural observations of the experimental material extracted from the actual turbine disk were carried out using an optical microscope (OM; Leica DM6000, Leica Microsystems Ltd., Mannheim, Germany) and a scanning electron microscope (SEM; JEOL-6010, JEOL Ltd., Tokyo, Japan) with an EDS detector. Stereology and quantitative metallography were adopted to evaluate the percentage of porosity according to the standard GB/T 15749-2008 [30]. OM images with magnifications of ×100 were collected and analyzed using Image-Pro Plus 4.1 software. The results show that the percentage of porosity of the samples is 0.0078%. The microstructure, displayed in the bright region of Figure 1a, contains a number of twins (indicated by black arrows) in the FGH96 superalloy. The γ’ phase is the main strengthening phase, and the volume fraction accounts for about 36% of the FGH96 superalloy. A small amount of large primary γ′ phase mainly distributed in the grain boundary on the specimen surface is clearly shown in Figure 1b.

**Figure 2:**
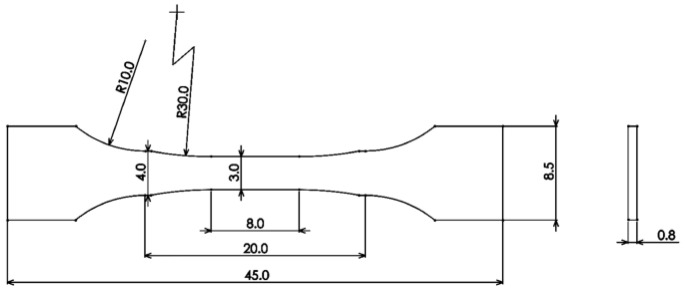
Dimensions of dog-bone-shaped specimen (in mm).

All fatigue test specimens with a gauge length of 45 mm and thickness of 0.8 mm were electrical-discharge-machined into dog-bone-shaped specimens. The schematic diagram of fatigue specimens is shown in Figure 2. According to standard GB/T 3075-2008 [31], the fatigue test specimens were precisely machined, and residual stress was minimized. Prior to the fatigue test, each side of the specimens was polished using SiC paper with grit scales ranging from 60 to 3000 and 1.0 μm diamond paste. Chemical corrosion was carefully administered for better observation. The maximum test stress selected in fatigue tests was 1100 MPa at room temperature (RT), and the stress ratio (R) was 0.1. All fatigue tests were conducted using a computerized INSTRON 8801 servo-hydraulic test machine at RT. During the whole cyclic loading process, the specimen surface was closely examined.

**Figure 3:**
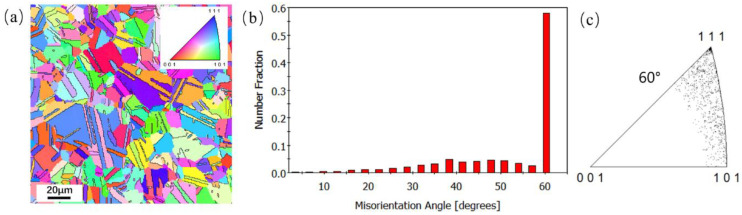
EBSD results of the FGH96 superalloy: (**a**) IPF map; (**b**) grain boundary orientation angle distribution diagram; (**c**) discrete plot of 60° grain boundaries.

An electron backscatter diffraction (EBSD) analysis using an Oxford Nordlys max3 was made to investigate the metallographic structure of the FGH96 superalloy (Figure 3). The EBSD data collected in this study were analyzed using TSL OIM 7.2 analysis software. The inverse pole figure (IPF) map in Figure 3a displays the high density of the annealing twins in the microstructure. The grain boundary orientation angle distribution diagram before the fatigue test is shown in Figure 3b, which demonstrates the distribution of the high-angle grain boundary of the FGH96 superalloy. According to the results of the discrete plot of 60° grain boundaries, nearly 60% of the total grain boundaries are parallel to the {111} plane and Σ3 twin boundaries (Figure 3c). In addition, EBSD analysis was also used to investigate the causes of crack deflection, which originated from pores in the fatigue test.

## 3. Results and Discussion

### 3.1. TIP Characterization

**Figure 4:**
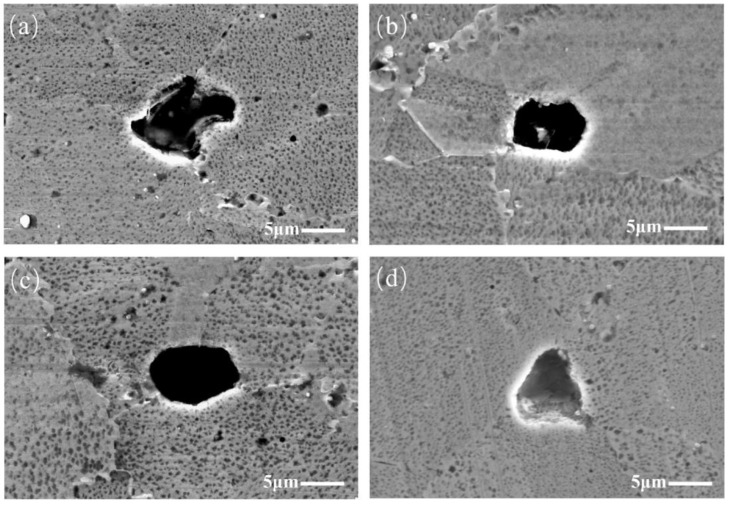
SEM images of isolated TIPs: (**a**) irregular shape; (**b**–**d**) regular shapes.

After the specimen was etched, the positions, shapes, and sizes of these TIPs in the alloy matrix were detected. It is worth noting that there are two types of TIPs. The first type of TIP is the isolated TIP, located at the grain boundary, as shown in Figure 4. Since all TIPs are located on grain boundaries, it is assumed that the formation of TIPs results from grain growth without much further densification during the consolidation process. Thus, the grains surround one other, leading to the formation of TIPs. It can be concluded that the configuration of TIPs located at grain boundaries is determined by grain growth during the consolidation process. Additionally, these isolated TIPs have either regular or irregular shapes. The regularly isolated TIPs typically exhibit a regular, two-dimensional shape, such as a circle, ellipse, square, and so on. Meanwhile, regularly shaped isolated TIPs exhibit greater symmetry. The shapes of the TIPs in Figure 4b–d are elliptic, elliptic, and triangular, respectively. Compared with the TIPs in Figure 4b–d, the symmetry of the isolated TIP in Figure 4a is relatively reduced, and it can be regarded as an irregularly shaped TIP.

**Figure 5:**
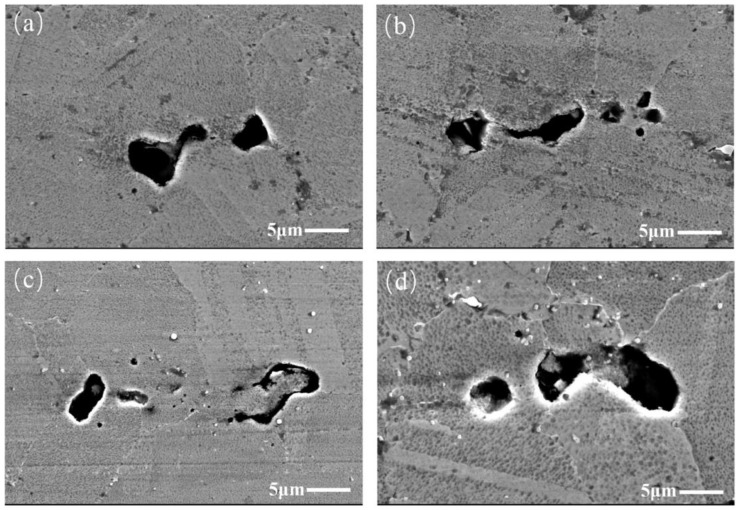
(**a**–**d**) SEM images of clustered TIPs.

The other kind of TIP is the clustered TIP. Clustered TIPs are composed of many unconnected small pores, and most of them are irregular in shape. Compared with isolated TIPs, clustered TIPs consist of a limited number of pores located within a limited grain boundary area, which is surrounded by pore-free grain boundaries. Most of these small pores exhibit a block-shaped morphology, while some of them are stretched along the grain boundary with diverse geometries, as depicted in Figure 5. The shapes of small, clustered pores that constitute the clustered TIPs appear to exhibit greater irregularity in comparison to isolated TIPs. The positions of the TIPs in the matrix were also inspected; they were generally located at the grain boundary.

**Figure 6:**
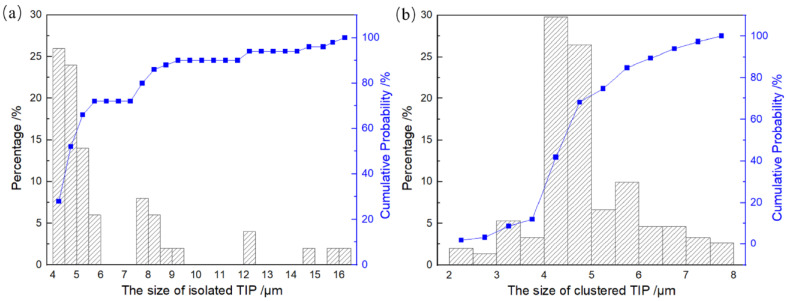
Size distribution and cumulative probability for two types of TIPs: (**a**) isolated TIP; (**b**) clustered TIP.

To calculate the size distribution of surface pores, it is necessary to characterize their sizes. A common method to calculate pore size involves measuring the diameter of a circle with an area equal to that of the pores in SEM and OM images of the surface. Image-Pro Plus 4.1 software was chosen for the estimation of the surface pore size. Note that the area of the clustered pores corresponds to the area of each of its individual constituent small pores, not the sum area of their areas. The size distribution and cumulative probability for two types of TIPs are shown in Figure 6. It can be observed that the sizes of isolated TIPs range from 4.0 to 16.5 μm, and approximately more than half of the pore size is in the range of 4.0 to 5.0 μm. Moreover, the size for clustered TIPs ranges from 2.0 to 8.0 μm, and approximately more than half of the pore size is in the range of 4.0 to 5.0 μm. The different size distributions of the small pores comprising the clustered TIPs preclude their classification as isolated TIPs. Consequently, a distinction between clustered TIPs and isolated TIPs needs to be made.

### 3.2. Crack Initiation at TIPs

#### 3.2.1. Crack Initiation at Isolated TIPs

The crack initiation of Ni-based superalloys is highly sensitive to surface defects such as machining marks and scratches, which result in surface roughening. Thus, the four sides of each specimen were manually polished using SiC paper with grit scales ranging from 60 to 3000, followed by a final polishing step using 1.0 μm pastes, in order to precisely control the surface roughness prior to conducting fatigue tests. Additionally, suitable chemical corrosion was utilized to facilitate fatigue inspection. The fatigue tests were performed at RT to prevent the deleterious effects of oxidation at high temperatures.

**Figure 7:**
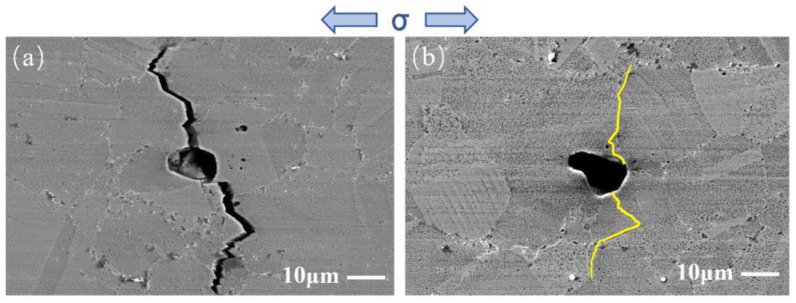
SEM images of crack initiation at isolated TIPs with different shapes: (**a**) regular circular shape; (**b**) irregular shape.

The procedure of crack initiation from isolated TIPs at RT is shown in Figure 7. The loading axis was parallel to the horizontal direction. As shown in Figure 7a, when the fatigue test was stopped at 88,166 cycles, a crack initiated from a regular circular pore with a crack length of approximately 58.1 μm. The diameter of the circular pore was approximately 13.3 µm. The observation result of crack initiation at an isolated TIP with an irregular shape is shown in Figure 7b. When the fatigue test was stopped at 45,379 cycles, an irregular pore initiated a crack with length of approximately 57.3 μm, and its equivalent circular diameter was approximately 15.1 µm. The crack initiation sites seem to be random. A crack initiated at a smaller-sized regular pore undergoes more cyclic loading times compared to a crack of nearly the same length initiated from an irregular pore. It is worth noting that too few loading cycles may lead to the absence of cracks on the surface of the specimen. Additionally, an excessive number of loading cycles will result in a larger crack opening distance, making it difficult to verify whether the cracks originated from surface pores. Therefore, it is imperative to carefully choose the observation cycle to ensure the surface cracks originated from surface pores. As a result, the fatigue tests were stopped at 88,166 cycles and 45,379 cycles, respectively, to ensure the observation of surface cracks attributed to surface pores, as depicted in Figure 7a,b.

**Figure 8:**
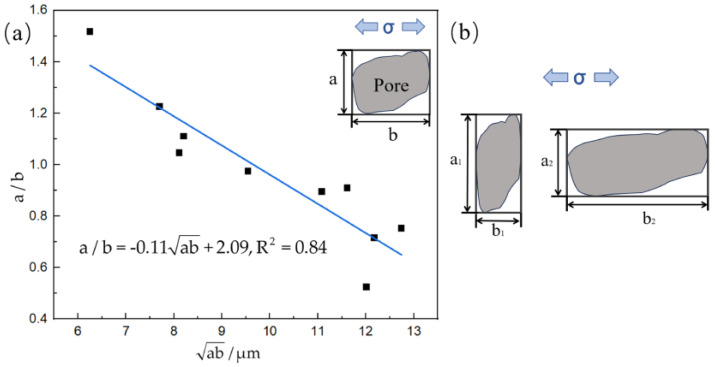
The relationship between the maximum equivalent isolated pore size and the ratio of the vertical−parallel axis: (**a**) the linear fitting results; (**b**) the schematic.

Accordingly, to provide a quantitative representation of isolated TIPs that initiate cracks, the maximum dimensions of the isolated TIPs in the vertical and parallel directions to the loading direction are defined by introducing axis length a and axis length b, respectively, as shown in Figure 8. Then, two parameters denoted by “a/b” and “$\sqrt{ab}$” are utilized to describe the maximum equivalent size and the ratio of the vertical–parallel axis of the isolated TIP, respectively. To carry out the calculation in Figure 8a, ten isolated TIPs with different shapes and sizes being origins of cracks were examined. A linear fitting method using Origin v2023 analysis software was introduced to investigate the correlation between the maximum equivalent pore size and the ratio of the vertical–parallel axis. The relationship between the maximum equivalent pore size and the ratio of the vertical–parallel axis can be approximately given using the following linear equation:

(1) $$a/b=-0.11\sqrt{ab}+2.09$$

where R^2^ is the coefficient of determination representing the reliability of linear fitting. R^2^ in the experimental results is 0.84, which means that the linear fitting can be considered sufficiently credible. It can be inferred from the results that the initiation behavior of the fatigue crack in the FGH96 superalloy with an isolated TIP is determined not only by the maximum equivalent size but also the ratio of the vertical–parallel axis. As depicted in Figure 8a,b, the ratio of the vertical–parallel axis of isolated TIPs that are the origins of cracks also shows a decreasing trend as the maximum equivalent size increases. The ratio of the vertical–parallel axis can be seen as the orientation of the pore relative to the loading direction. Regarding an isolated TIP with an equivalent size, a larger ratio means that the pore is more perpendicular to the loading direction, which will more easily initiate cracking. Moreover, the negative slope coefficient of the linear equation also proves that though the pore size is small, there is a large enough ratio of the vertical–parallel axis of an isolated TIP to likely initiate cracking.

#### 3.2.2. Crack Initiation at Clustered TIPs

**Figure 9:**
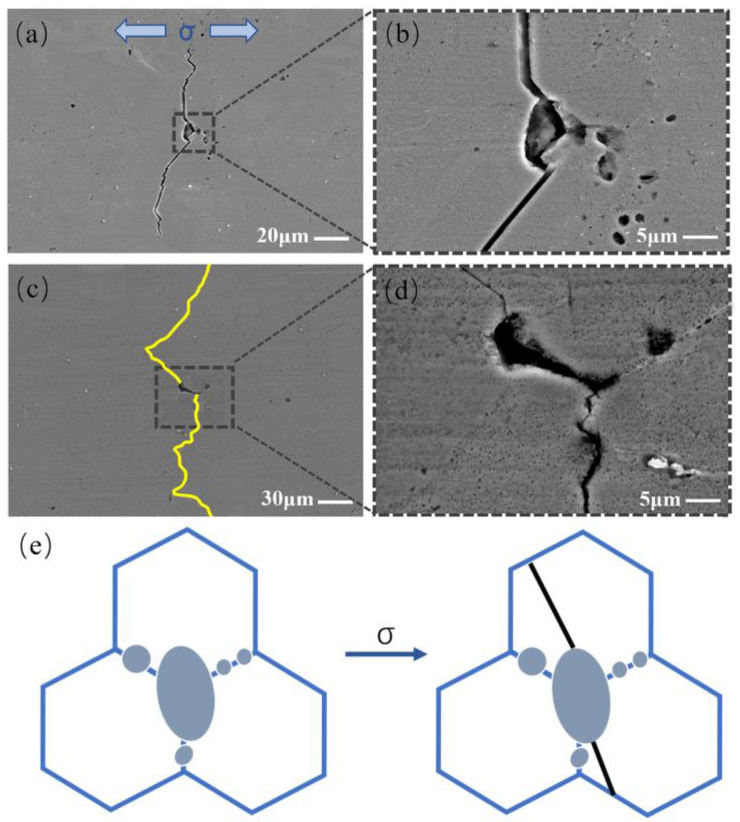
SEM images and schematic diagram of crack initiation at clustered TIP: (**a**–**d**) SEM images and partial enlarged images; (**e**) the schematic diagram.

Clustered TIPs are composed of many unconnected small pores of different sizes. The constituent pores of the clustered TIPs will compete to initiate cracks based on the experimental results. Figure 9 shows two cracks that originated from clustered TIPs. The two cracks both originated from the two largest pores in Figure 9b,d. Zhang et al. [32] previously pointed out that the final fracture can be caused by a larger pore. It can be speculated that for TIPs composed of different-sized small pores, the larger pores will be more likely to initiate cracks. The schematic diagram for crack initiation at clustered TIPs is displayed in Figure 9e. On the basis of the observation results, once the largest pore initiates cracking, other small pores around the largest pore will not have the chance to initiate cracking. The reason is that once a crack is initiated at the largest pore of clustered TIPs, the stress will be released immediately. As a consequence, other small pores will not have the chance to nucleate cracks. Under given experimental conditions, short fatigue cracking in the Ni-based superalloy mainly propagates in a transgranular manner. The constituent pores of the clustered TIPs located at the grain boundaries exhibit a random spatial distribution. There is also a notable distance between the constituent pores of the clustered TIPs. Consequently, the cracks initiated from clustered TIPs may not propagate through all the pores in the cluster.

#### 3.2.3. The Mechanism of Crack Initiation at TIP

**Figure 10:**
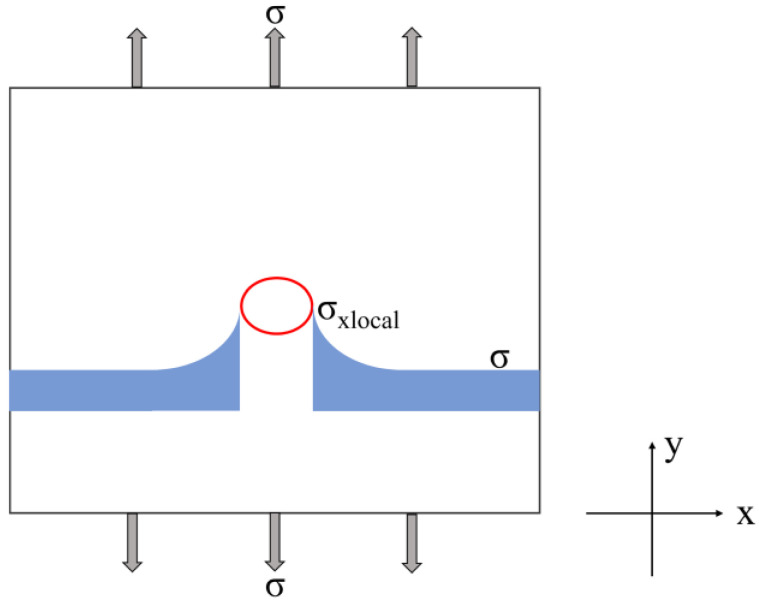
The diagram of stress distribution around the pore.

According to the observation results (Figure 4 and Figure 5), TIPs in the Ni-based superalloy generally locate at the grain boundaries. The TIPs exhibit both microstructural discontinuities and geometrical discontinuities in the material [33,34]. As shown in Figure 10, the stress at the root of the elliptical pore in an infinite plate has a higher value than the remote stress. It is noteworthy that the high stress surrounding the surface pores can lead to localized strain accumulation and plastic deformation under fatigue loading, which in turn can accelerate the initiation of fatigue cracks [35,36]. Consequently, the pores increase the chance of early initiation. Fatigue cracks will be more likely to originate from the root of the pores.

### 3.3. Crack Propagation

#### 3.3.1. Crack Propagation Observation

**Figure 11:**
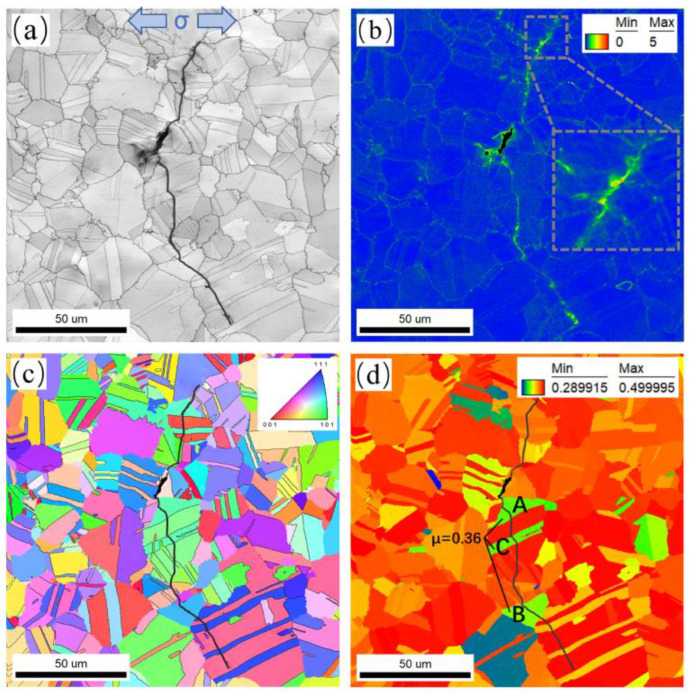
EBSD observations of crack 1: (**a**) IQ map; (**b**) KAM map; (**c**) IPF map; (**d**) SF map. A, B and C in subfigure (**d**) indicate grains A and B and twin C, respectively.

Figure 11 shows the EBSD observation results of crack 1, including an image quality (IQ) map, IPF map, kernel average misorientation (KAM) map, and Schmid factor (SF) map. As shown in Figure 11a, after over 126,985 cycles of loading, a crack originates from the pore in the central black area and propagates into the matrix with a length of about 146.4 μm. It is clear that crack 1 propagates in a transgranular manner at RT. Neither microcracks nor coarse grains are present around the crack tip, and the crack propagation is continuous. The KAM value at the tip of the crack is higher in Figure 11b, which demonstrates high dislocation density distribution areas at the tip of the crack. Additionally, a high KAM value at the crack tip is also associated with a high local plasticity and a large amount of dissipated energy for crack propagation at the early stage of crack development. The EBSD IPF map shows the grain orientation near crack 1 (Figure 11c). It is found that the short crack growth path has different degrees of deflection when passing through neighboring grains. Crack deflection can be caused by the inhomogeneity of the local grain orientation along the crack growth path when approaching grain and twin boundaries. Consequently, crack deflection consumes more energy for propagation, and a larger deflection angle means that more energy is consumed. As shown in Figure 11d, the SF distribution diagram illustrates that the color of the grain pattern through which the crack passes is predominantly orange and red. The grains with high SF and low SF are randomly distributed in the FGH96 superalloy. The SF value of green grains A and B and twin C is relatively low, measuring at 0.36, compared with adjacent grains. It is apparent that when the crack enters or passes through the grain boundaries and twin boundaries of grains A and B and twin C, a much larger crack deflection angle is observed. When the crack passes through green hard-orientation grains and twins, crack propagation could be hindered. The reason for this is that the sliding activation of the {111}<110> slip system is difficult to initiate in grains with a low SF, and the presence of grains with a low SF impedes crack propagation and largely changes the direction of crack propagation.

**Figure 12:**
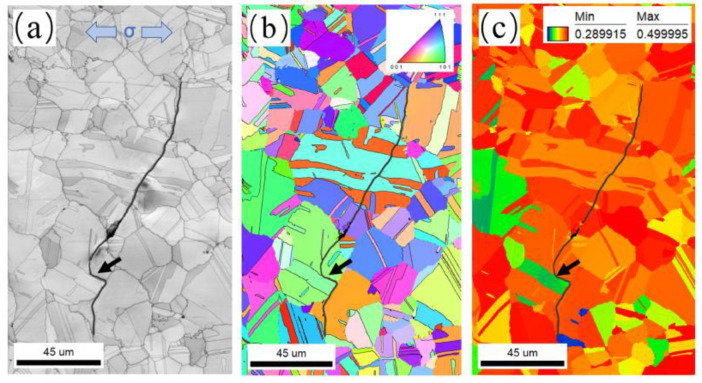
EBSD observations of crack 2: (**a**) IQ map; (**b**) IPF map; (**c**) SF map. The black arrows indicate the crack deflection position.

The propagation path of crack 2, which originated from a surface pore, was also observed. Figure 12 shows the EBSD results of crack 2 including IQ, IPF, and SF maps. As depicted in Figure 12, the crack propagates straight on top of the deflection position (indicated by the black arrow) when it passes through multiple grains. The SF value of these grains ranges from 0.45 to 0.48 on the top of the deflection position, implying that these grains are almost soft-orientation grains. The activation of slip systems in these grains with soft orientations is relatively easy to initiate under stress loading. As a result, the crack propagates in a straight line without a distinctive deflection.

**Figure 13:**
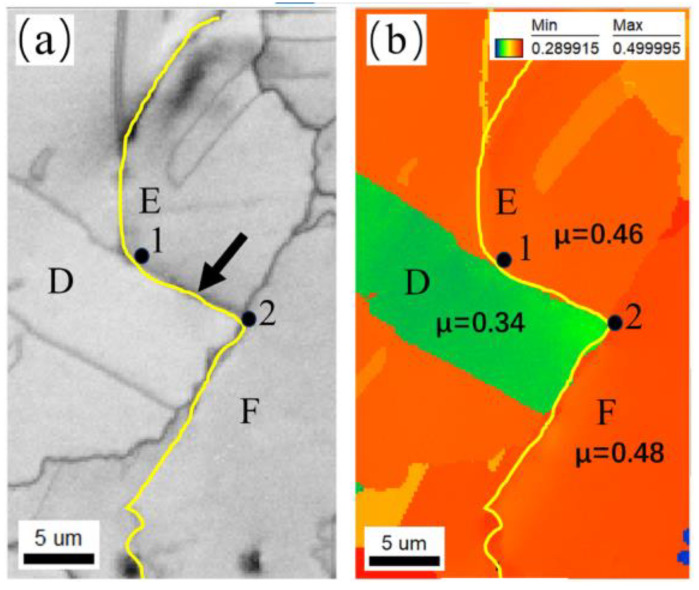
A partial enlarged image of the position indicated by the black arrow of crack 1 shown in Figure 12: (**a**) IQ map; (**b**) SF map. D, E and F indicate twin D, grains E and F, respectively. The black arrow indicates the twin boundary of twin D. Point 1 and 2 indicate the crack deflection position.

Figure 13 presents a partial enlarged image of the crack deflection position (indicated by a black arrow) shown in Figure 12. It can be observed that the location indicated by a black arrow is the twin boundary of twin D. When the crack propagates from grain E to intersect with twin D, the crack deflects at a large angle at point 1 and propagates along the twin boundary until it intersects with grain F at point 2. Next, the crack deflects at a large angle again at point 2 and then propagates along the grain boundary of grain F. Finally, the crack enters grain F. The reason for large-angle crack deflection is that the SF of twin D is 0.34, which is much lower than grains E (0.46) and F (0.48). Compared with grains E and F, twin D is less likely to activate the {111}<110> slip system under stress loading. As a consequence, the twin boundary of twin D with lower boundary energy becomes the preferred direction for crack propagation, leading to large-angle crack deflection. Then, a crack propagates along the grain boundary until it enters the grain with a higher SF and returns to transgranular manner.

#### 3.3.2. Crack Deflection Analysis

Grain boundaries are capable of dividing two adjacent grains with a similar structure but various orientations, which generally exist in crystalline materials. Twin boundaries, regarded as a typical type of grain boundary, also play a key role in metallic materials with different crystal structures. Based on the above consequences, it can be observed that a crack in the FGH96 superalloy mainly propagates in a transgranular manner at RT. Crack deviations and local deflection are constantly detected during crack propagation, especially when the crack is approaching grain boundaries and twin boundaries due to the inhomogeneity of the local grain orientation along the crack growth path. Therefore, both grain and twin boundaries can contribute to an increased crack propagation path length.

SF can be utilized to assess the plastic deformation ability of grains [37,38] and provide a rational explanation for fatigue crack propagation behavior. On the one hand, most of the grains for transgranular crack propagation have an SF larger than 0.4. This is in accordance with the phenomenon in René 88 DT mentioned in the literature [39]. On the other hand, the SF exhibits the ability to forecast the deflection of a short crack path, particularly at grains and twins with a low SF. As is well known, the activation of the slip system in grains with a low SF is difficult. Hard-orientation twins with an SF lower than 0.4 are more often associated with crack deflection. For example, when the SF of the twins in the FGH96 superalloy is much lower than that of the previous grain, it will be much harder to activate the slip system. As a result, the crack will not follow the original path to propagate through the twins in a straight direction; instead, it is deflected at a large angle before intersecting with the next grains. Sometimes, the crack may even propagate along the twin boundaries with lower grain boundary energy to avoid entering hard-orientation twins, which causes large-angle deflection. Then, the crack propagates along the grain boundary, which is perpendicular to the twin boundary, for a short distance in an intergranular manner. Finally, the crack enters the next grain with a high SF and eventually propagates back to the original transgranular manner. Generally speaking, large-angle crack deflection that consumes more energy for propagation will greatly increase the length of the crack propagation path and the resistance to crack propagation. Therefore, grains and twins with a low SF play a significant role in hindering fatigue crack propagation.

## 4. Conclusions

The effect of thermally induced pores on crack initiation in the FGH96 superalloy was examined under fatigue loading at RT. Some conclusions from the present work are detailed below:

1. There are two forms of thermally induced pores including isolated TIPs and clustered TIPs. The isolated TIPs have both regular and irregular shapes, and clustered TIPs have much more irregular shapes. Most of the TIPs are located at the grain boundary. The isolated TIPs range in size from 4.0 to 16.5 μm, and small pores, which clustered TIPs are composed of, range in size from 2.0 to 8.0 μm.
2. The initiation behavior of fatigue cracking in the FGH96 superalloy with isolated TIPs is determined not only by the maximum equivalent size but also by the ratio of the vertical–parallel axis to the loading direction of the pore. Regarding clustered TIPs, the constituent pores of the clustered TIPs will compete to initiate cracks based on the experimental results, and the largest pore will be more likely to initiate cracking.
3. Grains and twins with a low SF play a significant role in hindering fatigue crack propagation. Large-angle crack deflection, which consumes more energy, greatly increases the length of the crack propagation path and the resistance to crack propagation.

However, it must be recognized that further research is required, particularly focusing on the following themes:

1. Investigating the fatigue scatter of materials while considering the impacts of TIPs based on cumulative results—utilizing extreme value statistics would help in the investigation of the more detrimental forms of thermally induced pores.
2. Exploring the possible patterns and reasons for the formation of isolated-TIP and clustered-TIP turbine disks under different processing procedures.

## Figures and Tables

**Table 1:** The composition of the FGH96 superalloy (wt.%).

Cr	Co	Zr	Mo	Ta	W	Al	Ti	Nb	B	C	Ni
16.00	13.00	0.04	4.00	2.30	4.00	2.20	3.70	0.80	0.02	0.05	Bal.

**Table 2:** The mechanical properties of the FGH96 superalloy.

T [°C]	E [GPa]	σ_b_ [MPa]	σ_0.2_ [MPa]	υ
20	225	1546	1107	0.30

## Data Availability

The data presented in this study can be obtained upon request from the corresponding author.
